# Lab-on-chip analyser for the in situ determination of dissolved manganese in seawater

**DOI:** 10.1038/s41598-021-81779-3

**Published:** 2021-01-27

**Authors:** Felix Geißler, Eric P. Achterberg, Alexander D. Beaton, Mark J. Hopwood, Mario Esposito, Matt C. Mowlem, Douglas P. Connelly, Douglas Wallace

**Affiliations:** 1 Chemical Oceanography, Marine Biogeochemistry, GEOMAR Helmholtz Centre for Ocean Research Kiel, Kiel, Germany; 2 National Oceanography Centre, Southampton, SO14 3ZH UK; 3 Department of Oceanography, Dalhousie University, Halifax, NS Canada

**Keywords:** Biogeochemistry, Environmental monitoring, Marine chemistry

## Abstract

A spectrophotometric approach for quantification of dissolved manganese (DMn) with 1-(2-pyridylazo)-2-naphthol (PAN) has been adapted for in situ application in coastal and estuarine waters. The analyser uses a submersible microfluidic lab-on-chip device, with low power (~ 1.5 W) and reagent consumption (63 µL per sample). Laboratory characterization showed an absorption coefficient of 40,838 ± 1127 L⋅mol^−1^⋅cm^−1^ and a detection limit of 27 nM, determined for a 34.6 mm long optical detection cell. Laboratory tests showed that long-term stability of the PAN reagent was achieved by addition of 4% v/v of a non-ionic surfactant (Triton-X100). To suppress iron (Fe) interferences with the PAN reagent, the Fe(III) masking agents deferoxamine mesylate (DFO-B) or disodium 4,5-dihydroxy-1,3-benzenedisulfonate (Tiron) were added and their Fe masking efficiencies were investigated. The analyser was tested during a deployment over several weeks in Kiel Fjord (Germany), with successful acquisition of 215 in situ data points. The time series was in good agreement with DMn concentrations determined from discretely collected samples analysed via inductively coupled plasma mass spectrometry (ICP-MS), exhibiting a mean accuracy of 87% over the full deployment duration (with an accuracy of > 99% for certain periods) and clear correlations to key hydrographic parameters.

## Introduction

Manganese (Mn) is a redox sensitive trace metal and an essential micronutrient for marine phytoplankton^1^. Dissolved (< 0.45 µm) Mn (DMn), the most bioavailable size fraction, is essential for photosynthesis and may co-limit primary production in some parts of the Southern Ocean^2–4^. Manganese in its insoluble oxidized form (+ IV) plays an important role in the removal of other trace metals and rare earth elements from the water column due to adsorption processes onto Mn oxide particle surfaces^5^. Mn(IV) can be reduced to dissolved Mn(II) via photoreduction induced by sunlight^6^ leading to elevated DMn concentrations in the euphotic zone of open ocean waters, e.g. up to 2 nM in the West Atlantic^7^ and 3.2 nM in the East Atlantic Ocean^8^, where atmospheric dust deposition is the main Mn supply mechanism. Dissolved Mn concentrations of up to several tens to hundreds of nM can be found near sources such as hydrothermal vents^9,10^, and in coastal waters and fjordic systems as a result of Mn supply from reducing sediments and continental runoff^11,12^.

Conventionally, concentrations for Mn and other trace metals in natural waters are determined in discrete samples collected during surveys, followed by analysis in land-based laboratories using analytical techniques such as graphite furnace atomic absorption spectrometry (GFAAS)^13^, inductively coupled plasma mass spectrometry (ICP-MS)^14^ or inductively coupled plasma optical emission spectrometry (ICP-OES)^15^. A sufficient temporal and spatial resolution for environmental processes studies, on short timescales of seconds to days/weeks, therefore cannot easily be provided as this approach requires substantial logistical efforts with time delays of up to several months between sample collection and analysis. To resolve the highly variable DMn concentrations in marine systems, both in space and time, the traditional approach needs to be replaced by in situ techniques. Spectrophotometric methods can be easily simplified and miniaturized for in situ measurements using low cost components such as light emitting diodes (LEDs) and photodiodes (PDs). Utilized on a microfluidic lab-on-chip (LoC) based platform, spectrophotometry forms a powerful technique for robust and reliable in situ measurements, as demonstrated for nutrient and pH analysis^16–18^ up to a level ready for commercialization (with costs of about 15,000 € per unit). The cost of deploying such LoC analysers is far less than that associated with sample collection, handling and operation of ICP-instruments.

Lab-on-chip instruments facilitate all steps of a wet chemical colorimetric analysis from sampling, sample treatment, chemical reaction, detection and data processing on a single unit. The LoC analysers are characterized by low power (~ 1.5 W) and low reagent (in the µL range) consumption per measurement, a small size, portability, low limits of detection and ability for long-term deployments. Previously reported in situ DMn systems, e.g. the SCANNER^19^, SUAVE^20^, ZAPS^21^, GAMOS^22,23^ and METIS analysers^24^, have their individual advantages but suffer from some drawbacks. The analysers SCANNER, SUAVE and METIS use sensitive spectrophotometric approaches for the determination of Mn(II) in seawater, but have relatively high reagent and standard consumption (up to several mL/min) which limits their operational lifetime. The ZAPS probe and the GAMOS analyser provide good selectivity and sensitivity towards Mn(II) (down to sub-nanomolar concentrations) by utilizing fluorescence and chemiluminescence techniques, respectively. However, the ZAPS probe suffers from limited in situ calibration capability and requires extensive post-calibration efforts^21^. Despite the minimized fluid and power consumption of a second generation GAMOS analyser^23^ its portability is limited due to its physical size, based on the dimensions reported for the first generation GAMOS analyser^22^.

Several colorimetric methods are reported for Mn analysis in natural waters using different complexing agents, such as formaldoxime^25^, the porphyrin ligand T4CPP^26,27^ and 1-(2-pyridylazo)-2-naphthol (PAN)^28^. The formaldoxime method exhibits low sensitivity with a molar absorptivity of 10,700 L⋅mol^−1^⋅cm^−1^ at 450 nm^25^ along with poor linearity and cross-sensitivity to other cations^26^. In contrast, the method using the porphyrin ligand T4CPP provides high sensitivity and a fast reaction towards Mn(II) with a molar absorptivity of 95,400 L⋅mol^−1^⋅cm^−1^ at 468 nm^27^, but uses highly toxic Cd(II) compounds. The colorimetric method using PAN as complexing agent has found wide usage for Mn analysis as it features a fast reaction and high sensitivity towards Mn(II). The absorbance of the formed purple coloured Mn(PAN)_2_ complex is proportional to the Mn(II) concentration, with a high molar absorptivity of ~ 44,000 L⋅mol^−1^⋅cm^−1^ at the absorbance maximum of 562 nm^26,28^. This feature makes the PAN method ideally suited to be applied on a previously reported LoC device used for the quantification of iron (Fe) using the ferrozine method^29^ as also this colorimetric method exhibits its absorbance maximum at 562 nm. As the PAN reagent itself, and also the Mn(PAN)_2_ complex, are poorly water soluble, the addition of a non-ionic surfactant such as Triton-X100 is required to facilitate micelle formation and solubilize PAN and Mn(PAN)_2_ in the aqueous phase^28^. A key issue with colorimetric analysis is the potential for interferences. Addition of the Fe chelating reagent deferoxamine B (DFO-B) has been reported to suppress interferences from the presence of Fe(III), which is one potentially interfering ion in natural waters when using PAN^24,30,31^.

Here we evaluated the PAN method with respect to the reagent composition (use of Fe(III) masking agents, surfactants etc.) for in situ detection of DMn. The optimized method was then adapted for use in a microfluidic LoC analyser, which was deployed in coastal waters (Kiel Fjord, Germany) where moderately high Mn concentrations were anticipated from reducing sediments, freshwater runoff and possibly also anthropogenic inputs. Discrete samples analysed via ICP-MS throughout the deployment period served as a method validation tool.

## Results and discussion

### Characterization of PAN method

#### Mn(II) sensitivity

**Figure 1:**
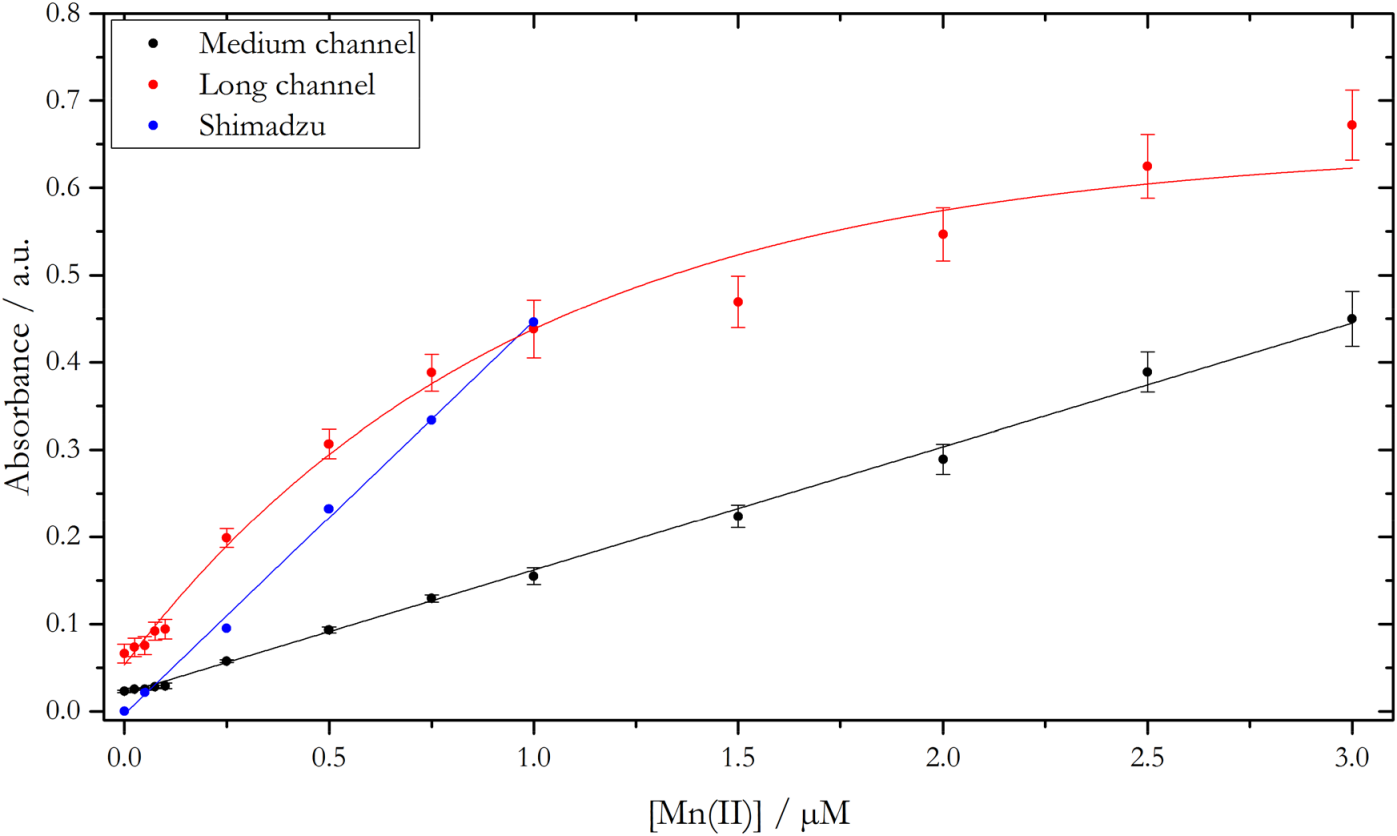
Calibration graphs for the detection of Mn using the PAN method (0.8 mM PAN in 4% v/v Triton-X100) acquired with the LoC analyser (long channel: 91.6 mm; medium channel: 34.6 mm; absorbance values were processed against a sample blank without the addition of PAN reagent) and a benchtop spectrophotometer (10 cm path length). Absorbance spectra from the spectrophotometer were measured against a reagent blank and processed at the absorption maximum of 562 nm.

The PAN method for the spectrophotometric detection of Mn was characterized with regard to its sensitivity and selectivity. Calibrations were obtained using both a double beam spectrophotometer (Shimadzu UV-1800) and the LoC device (Fig. 1).

Absorbance values obtained with the spectrophotometer (blue) and the medium channel of the LoC analyser (black) exhibited a linear relationship over the concentration range of 0–1 µM and 0–3 µM Mn(II), respectively. In contrast, absorbance values measured with the long channel of the LoC analyser (red) became non-linear above 0.5 µM Mn(II) (exponential fit shown in Fig. 1) due to too high absorbance values and therefore deviation from Beer–Lambert law and also exhibited a lower precision when measurements were repeated (e.g. for a 250 nM Mn(II) standard solution: R.S.D._Long channel_ = ± 5.4%, R.S.D._Medium channel_ = ± 2.8%; n = 20; error bars shown in Fig. 1). Therefore, the medium measurement channel was chosen for further experiments and in situ quantification of Mn(II). Linear slopes obtained with a benchtop spectrophotometer and the medium channel of the LoC analyser were distinct by a factor of ~ 3, with slopes of (4.503 ± 0.102) × 10^–4^ L⋅nmol^–1^ (R^2^ = 0.997) and (1.413 ± 0.039) × 10^–4^ L⋅nmol^−1^ (R^2^ = 0.991), respectively (Fig. 1), scaling with the different lengths of the optical paths, with a ~ 3 times longer path for the spectrophotometer (10 cm) than for the LoC analyser (3.46 cm). Normalized with respect to the length of the optical path, an absorption coefficient of 45,030 ± 1020 L·mol^−1^·cm^−1^ at 562 nm was obtained for the benchtop spectrophotometer, which is in good agreement with reported values of 44,000 L·mol^−1^·cm^−1^^28^ and 46,000 L·mol^−1^·cm^−1^^26^. For the medium measurement channel of the LoC analyser a somewhat lower absorption coefficient of 40,838 ± 1127 L·mol^−1^·cm^−1^ was obtained, which can be attributed to the difference between peak emission wavelength of 575 nm of the LED and the absorption maximum of 562 nm of the Mn(PAN)_2_ complex. However, the reported value here is still significantly higher than those reported for other in situ Mn analysers using the PAN method, such as for the METIS analyser with 8000 L·mol^−1^·cm^−1^^24^ and the SCANNER analyser with ~ 20,000 L·mol^−1^·cm^−1^ (estimated from calibration curve in Chin et al. (1992)^30^). The enhanced sensitivity of the LoC analyser here might be attributed to the high sample to reagent ratio of 8.8:1. In the METIS and SCANNER analysers the samples were mixed with reagent in a 1:1 ratio and a 5:1 ratio, respectively, resulting in reduced sensitivity. All presented values do not include any correction/compensation of the dilution factor, as the different dilutions are a characteristic feature of each individual device which determines the sensitivity. Additionally, the employed optical detection system (e.g. the LED emission spectrum and its overlap with the optical absorption peak of the Mn(PAN)_2_ complex) has an impact on the sensitivity. The detection limit (LOD) for the medium channel of the LoC device was 27 nM Mn(II), defined as three times the standard deviation of the analysis of a Mn(II) blank solution (n = 13) divided by the slope of the linear fit, and was within the LOD range reported for SCANNER (between 15 and 48 nM) but lower than the LOD of METIS (77 nM). The LoC analyser is therefore suitable for quantification of DMn concentrations in many freshwater bodies, coastal waters and near DMn sources such as hydrothermal vents, where elevated Mn concentrations of up to hundreds of nM can be found^9,12,32^.

#### Reagent composition

**Figure 2:**
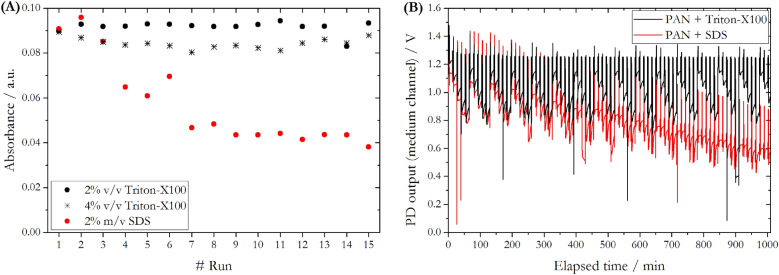
(**A**) Processed absorbance values of the analyser's medium channel of a 500 nM Mn standard solution analysed using three differently prepared 0.8 mM PAN reagents. (**B**) PD raw output from medium channel of LoC analyser for 15 consecutive runs of a calibration using four different Mn(II) standard solutions and 0.8 mM PAN reagent prepared with 2% v/v Triton-X100 and 2% m/v SDS.

As the PAN reagent and the Mn(PAN)_2_ chelate are poorly water soluble, a surfactant was added in order to allow micelle formation and solubilize both species in the aqueous phase. The PAN reagent prepared with 2% v/v of non-ionic surfactant Triton-X100 (as reported by Chin et al. (1992)^30^) was stable for 4 weeks. Visual examination showed precipitation of orange coloured PAN crystals when stored beyond 4 weeks. As any kind of particle formation needs to be avoided when using microfluidic technology, because of a high vulnerability to clogging in the narrow channels (e.g. 300 µm diameter), it was necessary to adapt the reagent composition with respect to the surfactant. Therefore, two different batches of PAN reagent were prepared; (a) with 2% m/v of the ionic surfactant SDS^24,33^ which facilitates faster reaction rates compared to Triton-X100 but lower molar absorbances^34^ and (b) with an increased concentration of 4% v/v of Triton-X100^33^ in order to provide a higher number of non-ionic surfactant molecules for micelle formation. Both reagents showed good stability as no particle formation was observable upon visual examination even after several months of storage, which is an essential requirement for long-term remote deployments. The response of the LoC analyser using reagents containing 2% v/v and 4% v/v Triton-X100 as well as 2% m/v SDS was tested (Fig. 2).

Processed absorbance values from the medium measurement channel were obtained for 15 consecutive analysis of a 500 nM Mn(II) standard solution with each reagent (Fig. 2A). For both reagents containing Triton-X100, absorbance values showed little variation (R.S.D. = ± 2.9%) indicating stable sensitivity over time. In case of the SDS surfactant, the absorbance decreased over time, resulting in a loss of sensitivity. Figure 2B indicates that the reduced sensitivity over time of PAN reagent prepared with SDS was linked to a decrease in the overall PD output, whereas a stable signal was obtained using PAN with Triton-X100. This may be caused by the SDS mediated formation of a light absorbing coating on the optical windows which separated the flow path from LEDs and PDs. Consistent with this explanation, rigorous flushing with 0.1 M sodium hydroxide solution brought the PD output back to the initial value. Sodium dodecyl sulphate forms a precipitation at temperatures < 15 °C, inhibiting its use in field-deployable systems for temperate and polar waters. Clinton-Bailey et al. (2017)^35^ suggested therefore the use of the dispersant polyvinylpyrrolidone (PVP) in microfluidic devices. However, PAN was not soluble in aqueous solutions containing PVP, even after stirring for one day at 80 °C. Therefore, the use of a PAN reagent containing 4% v/v Triton-X100 was chosen as optimum reagent solution for environmentally relevant conditions, ensuing stable sensitivity and no observable precipitation of PAN crystals over a period of several months.

#### Iron interference with PAN Mn analysis

The metals iron, zinc, nickel, copper and cobalt show significant interferences with the PAN method at a wavelength of 562 nm, when prevalent in a free ionic form at equimolar concentrations with Mn^30^. Therefore, masking agents with high affinity for those cations may be necessary in order to suppress potential interferences when analysing natural water samples. The siderophore type chelating agent DFO-B and catechol type ligand Tiron can bind these cations, with the highest affinity for Fe(III)^36,37^. DFO-B was used in several studies to date, as the strongest interferences are expected from Fe especially in natural waters with elevated Fe concentrations, such as near hydrothermal vents or in coastal systems^12,24,30^.

**Figure 3:**
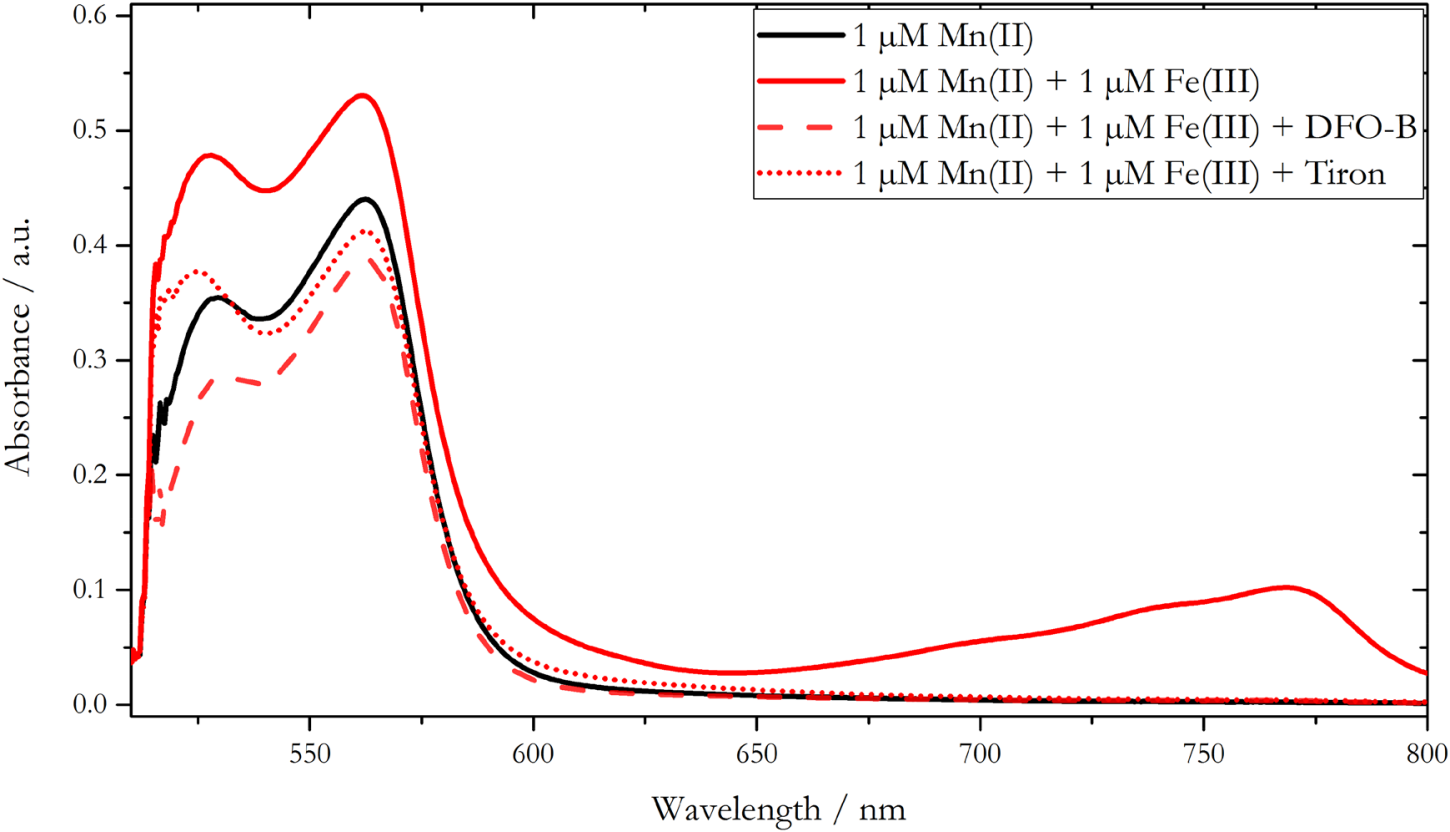
Absorbance spectra of a 1 µM Mn(II) standard solution and 1 µM Mn(II) solutions containing 1 µM Fe(III) with DFO-B and Tiron, added as Fe masking agents. All spectra were measured using 0.8 mM PAN reagent prepared with 2% v/v Triton-X100.

The spectrum obtained from a 1 µM Mn(II) + 1 µM Fe(III) standard solution (solid red, Fig. 3) featured a broad absorption band at wavelengths > 650 nm (with an absorption maximum at 768 nm) as well as an increased absorbance at 562 nm compared to the 1 µM Mn(II) standard solution (black, Fig. 3). The absorbance at 562 nm of the mixed Mn(II) and Fe(III) standard was equivalent to a processed Mn(II) concentration of 1.2 µM, and thus an Mn overestimation of 20%, which is in agreement with the value reported by Chin et al. (1992)^30^. With the use of DFO-B as Fe masking agent, the absorption spectrum of the mixed Mn(II) and Fe(III) standard (dashed line, Fig. 3) featured no absorption band at wavelength > 650 nm indicating efficient Fe(III) masking capability. However, the absorbance at 562 nm decreased compared to the 1 µM Mn(II) standard, resulting in a Mn(II) underestimation of ~ 15%. It appeared that the DFO-B removes part of the Mn(II) from the PAN accessible pool via DFO-B promoted oxidation to Mn(III) and subsequent stabilization of the higher oxidation state^38^. An underestimation of in situ determined DMn concentrations using the PAN method with DFO-B compared to ICP-MS measurements of discrete samples was also found for the METIS analyser^24^. Whilst this was ascribed to internal hardware issues rather than to DFO-B related issues, it may have been a combination of both based on our findings.

**Figure 4:**
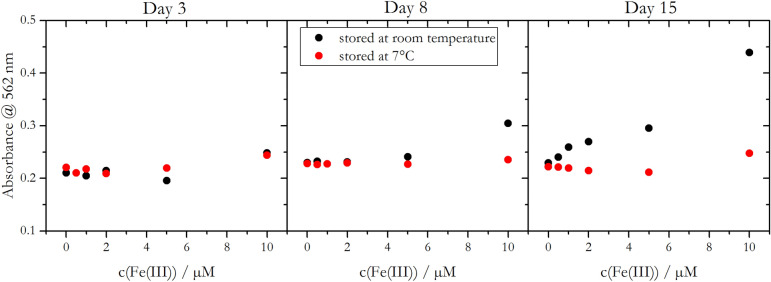
Absorbance at 562 nm of a 500 nM Mn(II) standard solution spiked with different amounts of Fe(III). Values were obtained after mixing standard solutions with two differently stored PAN/DFO-B reagents (at room temperature—black, at 7 °C—red) 3, 8 and 15 days after preparation. The two mixed PAN reagents contained 0.8 mM PAN and 400 µM DFO-B.

It was further observed that the Fe masking strength of DFO-B decreased over time, when stored as mixed PAN/DFO-B reagent at room temperature (Fig. 4). Three days after the preparation of the mixed PAN/DFO-B reagent, any Fe(III) interference was effectively suppressed, also when Fe(III) was present in large excess (up to 20 times of the Mn(II) concentration) in a 500 nM Mn(II) standard solution, for reagents stored at room temperature (black) and at 7 °C (red). The measured absorbance values were comparable to a standard solution without Fe(III) addition. Increased absorbance values were observed for the analysis of standards containing 5 µM and 10 µM Fe(III) after storage for 8 days at room temperature, and degradation of DFO-B led to observable Fe interferences beyond 8 days even at equimolar concentrations of Mn(II) and Fe(III) (500 nM of each). Storage at lower temperatures impeded the degradation of DFO-B and up to 5 µM Fe(III) was effectively masked even 15 days after reagent preparation.

Similarly to DFO-B, the catechol type Fe complexing agent Tiron showed effective Fe masking capability. No absorption band was observable at wavelength > 650 nm (Fig. 3, dotted line). However, a lower absorbance at 562 nm was found for the 1 µM Mn(II) + 1 µM Fe(III) standard solution when applying Tiron compared to the 1 µM Mn(II) standard analysed without Tiron. This suggests that Tiron also complexes Mn(II), resulting in an underestimation of Mn(II).

These findings suggest that DFO-B and Tiron have drawbacks as masking agent for in situ determination of Mn(II) using the PAN method; because of (1) a potential underestimation of Mn(II) and (2) the limited life time of DFO-B. Degradation might be a particular problem for remote in situ deployments over extended periods of weeks to months. However, in most natural waters, such as the Kiel Fjord, the metals which are interfering with the PAN method occur at low concentrations compared to Mn(II) (see next section). Furthermore, these trace metals are naturally prevalent in seawater as strongly bound organic complexes^39,40^, which limits their availability to form a complex with PAN reagent over short equilibration time periods^30,41^. With this critical evaluation we justify the application of a measurement protocol without any Fe masking agent at low Fe(III) concentrations as this would be linked to an underestimation of DMn. Therefore, we deployed the LoC analyser in an estuary, Kiel Fjord, without addition of a masking agent to the PAN reagent and evaluated the outcomes through LoC measurement validation against ICP-MS.

### In situ time series in Kiel Fjord

#### Hydrographic setting

**Figure 5:**
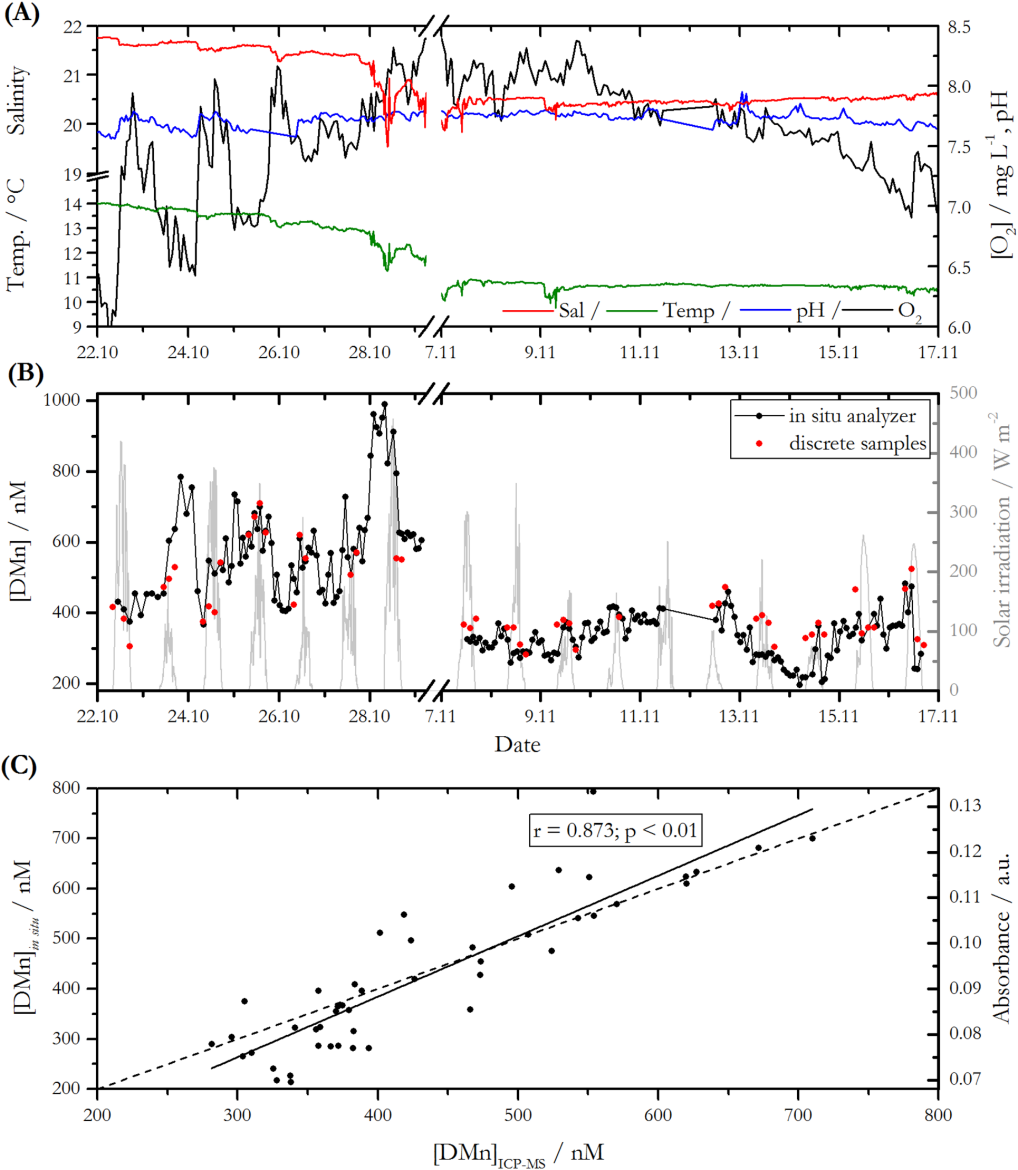
Time series for the periods October 22 to 29, 2018, and November 07 to 17, 2018, of (**A**) in situ determined hydrographic data and (**B**) DMn time series determined with in situ LoC analyser (black) and from discrete samples analysed via ICP-MS (red). (**C**) Scatter plot of in situ absorbance and DMn concentration vs. ICP-MS analysis (linear fit: [DMn]_in situ_ = 1.207⋅[DMn]_ICP-MS_ – 98.310; Spearman correlation parameters: r = 0.873, p = 1.3⋅10^–15^, n = 47); y = x (dashed line) is also displayed for clarity.

The performance of the DMn in situ analyser was evaluated during a deployment in the Kiel Fjord from October 22 to November 17, 2018. Kiel Fjord forms part of the southwestern Baltic Sea, and is subject to anthropogenic perturbations due to shipping and shipbuilding activities and discharges from a population of ca. 250,000 in the surrounding area. The main sources of freshwater input include rainwater run-off from the city of Kiel, the Schwentine River which is located at the eastern shore of the inner Kiel Fjord, and the Kiel-Canal which represents one of the busiest artificial waterways worldwide, located at the western shore of the fjord. The Kiel Fjord has a mean depth of ~ 13 m, with water level changes of up to ± 1 m caused by winds and pressure gradients over the Baltic Sea. During the period of the deployment, a storm flood approached Kiel Bay in two phases with a water level rise of up to 0.7 m on October 27 and up to 1.0 m on October 29 due to strong northerly winds. This hydrological extreme event caused reductions in salinity of 1.5 and in water temperature of 1.5 °C within less than 12 h (Fig. 5A). Before and after the storm flood, salinity and temperature were almost constant with a mean salinity of 21.5 ± 0.1 and a mean temperature of 13.5 ± 0.3 °C before the flood (October 22–27), and a mean salinity of 20.4 ± 0.1 and a mean temperature of 10.7 ± 0.1 °C after the flood (November 07 to 16). A minor response of pH and oxygen concentration to flood conditions was observed over the same time period. During the period of the deployment, mean pH values of 7.74 ± 0.10 and oxygen concentrations of 7.9 ± 0.9 mg⋅L^−1^ were observed. Analysis of discrete samples for nitrate, phosphate and DOC showed mean concentrations of 2.1 ± 0.7 µM, 1.3 ± 0.1 µM and 257 ± 15 µM, respectively, over the time period October 22 to November 17, 2018.

#### Performance evaluation of the analyser

During the period October 29 to November 7 the system underperformed, producing unreliable data for both the on-board standard solutions and the natural water samples, with negative calibration slopes and a relative change of up to 70% between adjacent time points, respectively. This period was therefore excluded from the time series shown in Fig. 5. Replacement of the filter attached to the sample inlet and a careful cleaning procedure of the microfluidic manifold with detergent and de-ionized water resolved the issues and therefore we assume that the fault was caused by a clogged filter membrane and/or microfluidic channels as a consequence of high suspended material loads in the water column during the storm flood event.

**Figure 6:**
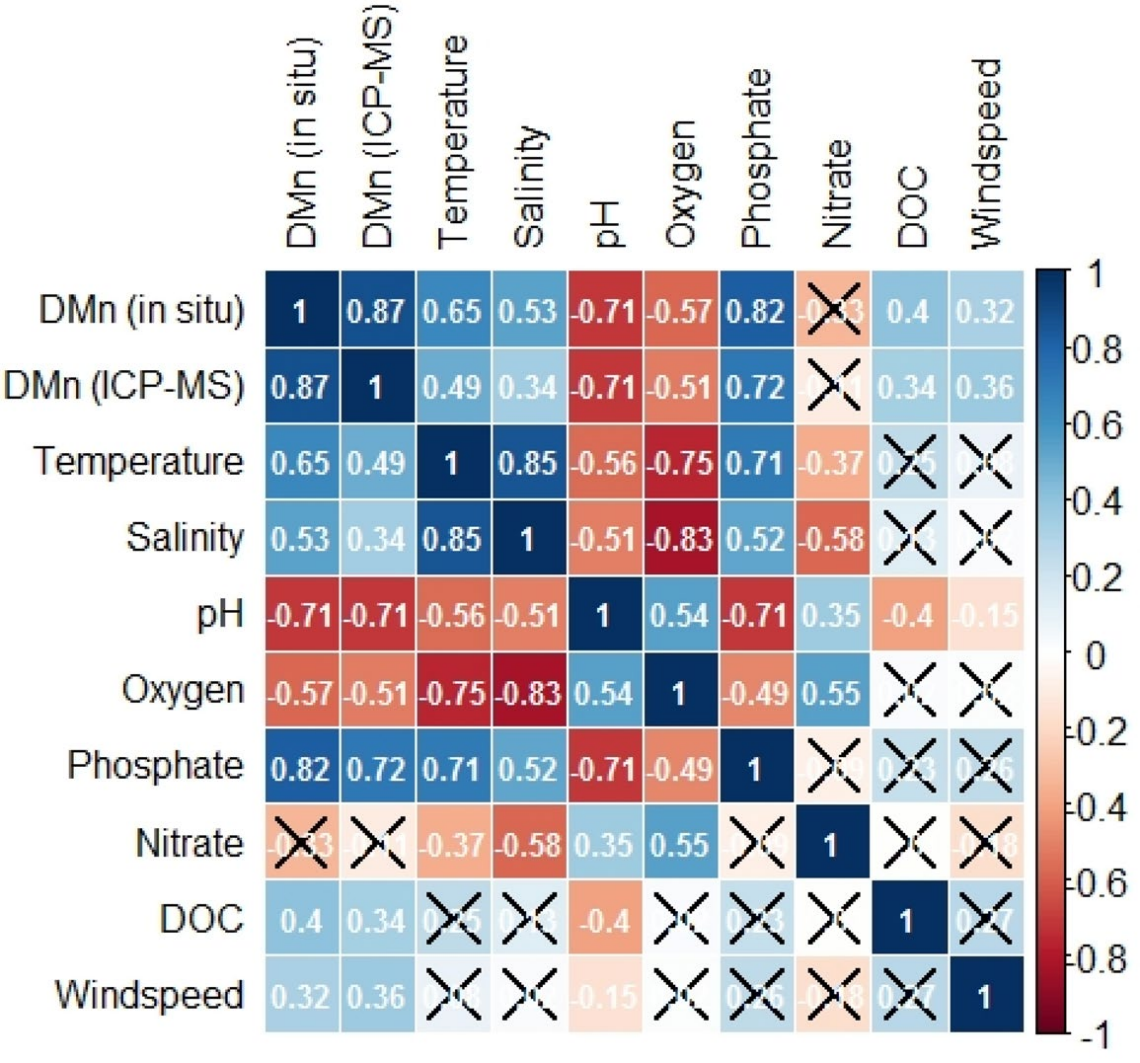
Spearman correlation matrix of in situ and discretely determined variables calculated with data points from October 22 to November 17, 2018; in situ DMn data for the period October 29 to November 07 were excluded for statistical analysis. Positive correlation coefficients are illustrated in blue boxes, negative ones in red. Not statistically significant correlations (p value ≥ 0.01) are crossed.

Reliable absorbance values for natural water samples mixed with the PAN reagent, with a relative change of less than 35% between adjacent time points, were obtained by the analyser for the periods October 22 to 29 and November 7 to 17. The analysis of on-board blank and standards for the purpose of in situ calibration produced highly variable calibration values, likely due to problems with their supply towards the microfluidic manifold. Slopes ranging from − 1.307 × 10^–4^ L·nmol^−1^ to 4.607 × 10^–4^ L·nmol^−1^ (mean: (1.955 ± 0.751) × 10^–4^ L·nmol^−1^) and intercepts in the range from 0.011 to 0.123 (mean 0.038 ± 0.020) were obtained in situ. Therefore, absorbance values of the natural water sample were processed using laboratory calibration of the analyser with standards prepared at a salinity of 18 (i.e. calibration curve slope = 1.104 × 10^–4^ L·nmol^−1^/intercept = 0.046). Due to a temperature offset of ca. 10 °C between laboratory calibration (conducted at 20 °C) and in situ temperatures, calibrations were conducted at 10 °C, 15 °C, 20 °C and 25 °C using a benchtop spectrophotometer equipped with a temperature-controlled cuvette holder. No change of sensitivity was observed when different temperatures were applied. DMn concentrations of 215 in situ measurements and 47 discrete samples analysed via ICP-MS as validation method are presented in Fig. 5B. Considering the data points of both time series with matching time stamps (n = 47) a mean accuracy of 87% was achieved for in situ measurements compared to ICP-MS samples, resulting in a strong significant correlation with a Spearman correlation coefficient of 0.873 (p < 0.01, n = 47; see Figs. 5C and 6). Particularly during the period October 24 to 27 DMn concentrations determined with the two methods were in close agreement with a high accuracy of > 99%. Between November 8 and 13, a mean offset of < 6% between the in situ determined concentrations compared to discrete samples was observed. A comparison to ICP-MS determined DMn concentration is more robust than comparison to samples treated with PAN reagent and analysed via a benchtop spectrophotometer. This verifies not only that the analyser measurements were reproducible, but also that sensor derived measurements were not subject to interferences or low recoveries—which it would not be possible to determine directly from benchtop measurements using the PAN method. In prior work using an earlier version of the analyser it was verified that the sensor derived measurements were approximately the same as benchtop measurements^31^, but as noted this only demonstrates the correct mechanical functioning of the sensor and not the accuracy of DMn concentrations.

Considering all data points (Fig. 5B), DMn concentrations determined in the discrete samples were in between 282 and 710 nM, with a mean DMn concentration of 430 ± 104 nM (n = 52). Using the in situ LoC analyser, a minimum of 196 nM and a maximum of 990 nM were observed, with a mean DMn concentration of 435 ± 163 nM (n = 215), in good overall agreement with the value obtained from discrete samples. However, as shown in Fig. 5B, there were several periods where in situ determined DMn concentrations were either lower or higher compared to discrete samples. This was also indicated by the divergent extreme values of both time series. For example, distinct overestimation of 18 ± 12% on average was observed from October 22 to 24, whereas the period November 12 to 16 was characterized by 17 ± 14% on average lower in situ DMn concentrations compared to those of discretely collected samples. The discrepancies between the two time series might be attributed to three facts: (1) analysis of slightly different waters due to the used deployment/sampling setup, (2) presence of Mn(III) species in the dissolved phase which was detectable with ICP-MS but not spectrophotometrically with the PAN method and (3) a lower temporal resolution of manually collected samples which may have missed a concentration peak observed by the analyser.

As to (1), the sample inlet of the LoC analyser was orientated horizontally at a water depth of ca. 2.0 m. Prior to each in situ analysis, 2.8 mL of the fjord water were aspirated for flushing of the channels. In contrast, the inlet of the hose used for collecting discrete samples was orientated vertically towards the seabed at the same depth as the analyser’s inlet, but 1.5 m laterally apart. Approximately 10 L of fjord water were pumped at a flow rate of ca. 1.5 L⋅min^−1^ through the system prior to sample collection to ensure a careful flushing of the hose and the attached filter cartridge. Due to the different orientation of the water inlet as well as a 3000 times higher water throughput for flushing purposes, it is possible that water from greater depths was aspirated with the setup for the discrete sample collection. Differences in DMn concentrations depending on the sampling depth may arise because of stratification of the water column, a decrease of light driven redox processes with depth (photo-enhanced reduction of high-valent Mn to the dissolved Mn(II) fraction^42^) and/or external DMn input to the upper layers e.g. through wet deposition.

As to (2), in contrast to the speciation of Fe in seawater, where the presence of organic ligands plays an important role, there is only limited evidence for organic complexation of Mn in seawater^43^. However, a recent study suggested the presence of organically complexed and therefore stabilized Mn(III) species by humic ligands in the DMn pool of estuarine waters^44^. Speciation studies showed that 17.6% of the DMn pool occurred as complexed Mn(III) species at 3 m depth, with increasing relative concentrations with depth in the St. Lawrence Estuary. Critically, those species are within the ICP-MS detectable DMn pool, whereas the spectrophotometrically detectable DMn pool, using the PAN method, accounts for only soluble Mn(II). Thus, the presence of dissolved Mn(III) species may have caused lower spectrophotometrically determined in situ DMn concentrations compared to those from discrete samples, e.g. from November 13 onwards (Fig. 5B).

As to (3), the variability in DMn concentrations between October 27 and 29 highlighted the need for temporally well resolved data, achievable only with in situ measurements. The observed dynamic nature of DMn concentration, with an increase of about 400 nM DMn during the night of October 27 to 28, was considered to be caused by the extreme weather conditions as the storm flood that approached the Kiel Bay was linked to an inflow of fresh water (drop of salinity of 1.5 PSU), a water level rise of up to 1 m a.s.l. and wind speeds of up to 13 m⋅s^−1^. This fast dynamic environment could not be resolved with discrete samples as, especially under those extreme weather conditions, sample collection was inconvenient and hazardous.

Overestimation due to the cross-sensitivity of the PAN reagent to other ions was negligible as all interfering ions occurred at low dissolved concentrations compared to DMn, with mean values of 90.8 nM Fe, 47.1 nM Zn, 15.0 nM Cu, 6.1 nM Ni and 0.7 nM Co, quantified from discrete samples via ICP-MS. Additionally, no statistically significant evidence of a correlation between the time series of these elements and an enhanced DMn fraction was found.

According to a Spearman rank correlation test, DMn concentrations showed significant relationships with a range of other parameters (Fig. 6; for time series see Fig. 5A,B). For example, in situ DMn concentrations anti-correlated significantly with oxygen and pH, with correlation coefficients of − 0.57 and − 0.71. The transformation between Mn(IV) oxides and dissolved Mn(II) species can be described as follows:

1 $${\text{Mn}}^{2+}+\frac{1}{2}{{\text{O}}_{2}+{\text{ H}}}_{2}{\text{O }}\rightleftarrows {\text{MnO}}_{2}+{2{\text{H}}}^{+}$$

Oxidation, and therefore a decrease of dissolved Mn(II) species, is favoured at increased oxygen concentrations and high pH values. In contrast, reduction of Mn(VI) oxides towards Mn(II) takes place under oxygen depletion and low pH values, typically in anoxic sediments with subsequent diffusion into overlying waters^45^. In natural waters, the reduction pathway is sun-light mediated, which is responsible for elevated DMn concentrations in the euphotic zone^6^. Furthermore, organic matter can mediate the (photo)-chemical reduction of Mn oxides to soluble Mn(II)^6^, which would explain the correlation between observed in situ determined DMn and DOC concentrations (r = 0.40).

A positive correlation with a Spearman correlation coefficient of 0.53 between DMn and salinity was found. This may be related to the storm flood, as we would generally expect an increase of DMn with decreasing salinity because freshwater, e.g. through riverine input, represents a major source of DMn species. When considering the time phases before and after the flood event individually, anti-correlations between DMn and salinity were found with correlation coefficients of − 0.27 and − 0.30, respectively. A strong statistical correlation was found between DMn and phosphate concentration (r = 0.82), suggesting that the marine biogeochemical pathways of both species are, together with oxygen, connected. In oxygenated waters, DMn species are oxidized towards Mn(IV) oxides (Eq. (1)). Mn(IV) oxides act as important adsorbents of phosphate, which leads to a removal of phosphate from the dissolved phase^46^. Upon reduction of particulate Mn in sediments, Mn(II) and phosphate are remobilized^45^.

This study demonstrated that LoC technology forms a powerful tool for in situ quantification of DMn species in a wide range of environments with respect to the linear detection range, e.g. in coastal waters and estuaries, in benthic boundary waters and in the vicinity of hydrothermal vents. Compared to previously reported in situ analysers which used the PAN method for the quantification of DMn in natural waters (e.g. SCANNER^30^, METIS^24^), a prolonged operational lifetime due to minimized fluid consumption and a higher sensitivity in terms of molar absorptivity was shown. Miniaturization led to an easily deployable device when compared to a modular system such as the SCANNER. Furthermore, the chemical recipe of the PAN reagent was adapted in order to improve its long-term stability and the effect of masking agents on the measurements was critically evaluated. The second generation LoC presented herein is a significantly updated version to that described by Milani et al. (2015)^31^, and features smaller microfluidic channels (160 × 300 µm vs 300 × 400/600 µm), lower average power consumption (1.5 W vs 3.8 W), improved electronics and software (Windows-based graphical user interface), and onboard data processing in order to acquire reliable and well resolved in situ DMn data over long periods most preferably remotely and unattended. This was demonstrated in the presented study with a deployment over 17 days, while the first generation LoC analyser as well as the METIS analyser were deployed on CTD casts where physical inspection and troubleshooting of the systems were facilitated between casts. The time series highlights the ability of LoC analysers to produce temporally well resolved measurements of trace metals, achievable only with in situ systems. This enables the unravelling of biogeochemical questions in remote areas, in dynamic areas with highly variable DMn concentrations over short time scales, or during hydrologically extreme events.

## Methods and materials

### Preparation of standard and reagent solutions

A thorough cleaning procedure was applied to all glass and plastic ware for standard and reagent preparation prior to their use. Soaking in a ~ 2% v/v acidic detergent bath (Citranox, Sigma-Aldrich) was followed by a 1.2 M HCl bath (reagent grade, Carl-Roth) for > 24 h. Glass and plastic ware was rinsed thoroughly with de-ionized water (MilliQ, 18.2 MΩcm, Merck Millipore) after each treatment and stored in plastic bags until required.

A working stock solution containing 100 µM Mn(II) was prepared on a weekly basis using 546 µL of a 1000 mg⋅L^−1^ Mn standard (1000 ppm Manganese for ICP, Inorganic Ventures) diluted to 100 mL with de-ionized water. Standard Mn(II) solutions were obtained by further dilution of the 100 µM Mn(II) working stock with de-ionized water. All Mn(II) solutions were stored at room temperature in opaque low-density polyethylene (LDPE) bottles (Nalgene).

The preparation of the PAN reagent followed, with some adaptations, the method reported by Chin et al. (1992)^30^. 0.05 g of 1-(2-Pyridylazo)-2-naphthol (PAN) (general purpose grade, Fisher Scientific) and 5 mL Triton-X100 (laboratory grade, Sigma-Aldrich) were added to approximately 50 mL of de-ionized water and stirred at 80 °C for at least 12 h until the PAN was dissolved completely. The warm orange coloured mixture was then poured into 100 mL of a 0.1 M borate buffer (pH ~ 10) and made up to 250 mL with de-ionized water, giving final PAN and Triton-X100 concentrations of 0.8 mM and 2% v/v (equivalent to 33 mM), respectively. For method evaluation, further PAN reagents were prepared containing 4% v/v Triton-X100 by the addition of 10 mL Triton-X100 or 2% m/v of the ionic surfactant sodium dodecyl sulphate (SDS) by the addition of 5 g SDS (ultra-pure, Carl Roth). No heat was required for the dispersion made with SDS to solubilize PAN. Borate buffer contained 0.618 g H_3_BO_3_ (99.99%, trace metal basis, Acros Organics) and 0.4 g NaOH (98.5%, Acros Organics) made up to 100 mL with de-ionized water, giving a concentration of 0.1 M for both boric acid and sodium hydroxide. As an interference of Fe(III) ions was reported for the PAN method^30^, two different Fe specific complexing/masking agents were tested: DFO-B and disodium 4,5-dihydroxy-1,3-benzenedisulfonate (Tiron). A 1.5 mM DFO-B solution was prepared by dissolving 5 mg of deferoxamine mesylate salt (95%, Acros Organics) in 5 mL de-ionized water. This solution was kept refrigerated when not in use. A 50 mM Tiron stock solution was prepared by dissolving 0.785 g of Tiron (Acros Organics) in 50 mL de-ionized water.

### Spectrophotometric benchtop experiments

For benchtop experiments using the PAN method, Mn(II) standard solutions and PAN reagent were mixed using a volumetric ratio of 9:1. For Fe(III) interference studies, the prepared Mn(II) standards were spiked with an acidified 100 µM FeCl_3_ solution (≥ 98%, Carl Roth) and a Fe(III) masking agent if required. Absorbance spectra between 400 and 800 nm were acquired after 5 min with a double beam Shimadzu UV-1800 spectrophotometer using 10 cm quartz cuvettes. Absorbance was processed at the peak maximum of 562 nm, and also at 768 nm for the Fe(III) interference experiments.

### Lab-on-chip analyser

**Figure 7:**
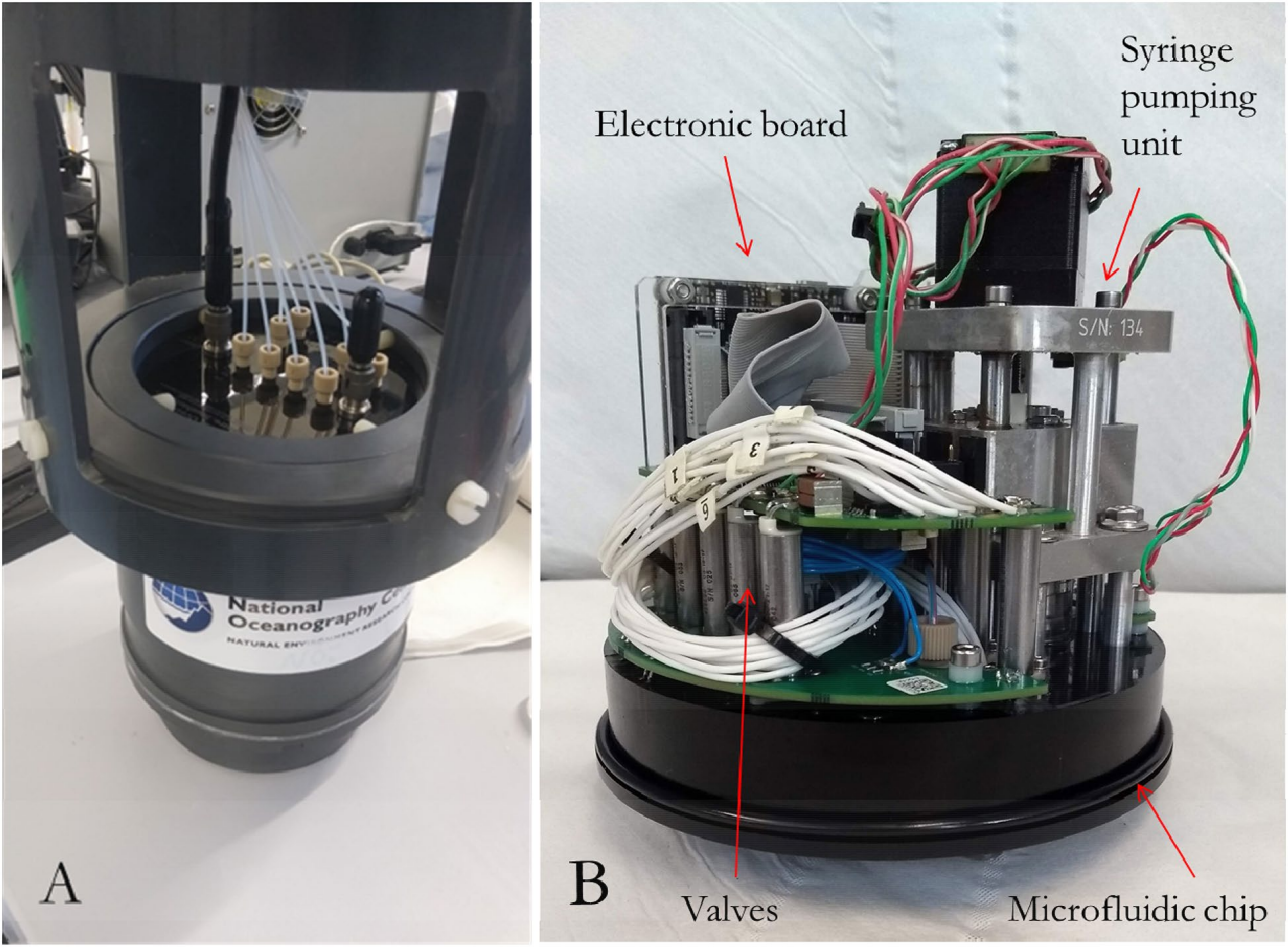
Hardware of second generation LoC analyser, (**A**) watertight PVC housing with microfluidic chip and fluid connectors, (**B**) microfluidic PMMA chip (119 mm in diameter) mechanical and electronic package (note the different orientation when mounted in PVC housing).

The PAN method was adapted for its application in a microfluidic LoC analyser. The analyser hardware (Fig. 7) was identical to a second generation device characterized for the detection of Fe in coastal waters using the Ferrozine (FZ) method^29^ as the Mn(PAN)_2_ complex features the same absorbance maximum at a wavelength of 562 nm as the Fe(FZ)_3_ complex. A first generation device was previously reported in Milani et al. (2015)^31^.

Briefly, the second generation analyser comprised a microfluidic chip (119 mm in diameter and 24 mm in thickness; Fig. 7B) made from tinted Poly(methylmethacrylate) (PMMA)^47^, with milled microfluidic channels of the dimensions 160 × 300 µm, forming the end-cap of a watertight polyvinylchloride (PVC) housing (Fig. 7A). A custom-built syringe pump unit including a stepper motor, two barrels for reagent supply (3.28 mm ID) and one barrel for sample and standard solutions (9.71 mm ID) was mounted onto the microfluidic chip for sample and reagent withdrawal from the reservoirs and injection into the microfluidic channels. Micro-inert solenoid valves (LFNA1250125H, The Lee Company) provided full fluidic control via individual actuation. Sample and standard solutions were mixed on-chip with the PAN reagent in a volumetric ratio of 8.8:1, defined by the volume of the barrels as the plungers of the pumping unit are moving simultaneously at the same speed. Here a waiting period of 15 min under stopped flow condition was sufficient to provide complete mixing and full colour development, leading to a maximum sample throughput of ~ 3 samples per hour including the flushing routine. After chemical reaction and full colour development the absorbance was measured in three individual optical channels of different length (91.6 mm, 34.6 mm and 2.5 mm) using LEDs with a peak wavelength of 575 nm (AlGaInP, B5B-433-20 LED, Roithner LaserTechnik GmbH) as a light source at the beginning of each optical cell, and PDs (TSLG257-LF, TAOS) as detection units for the transmitted light at the end of each optical channel. Three different path lengths were implemented in order to cover a broad linear detection range according to Beer–Lambert law in terms of analyte concentration, as demonstrated in Geißler et al. (2017). Monitoring PDs were mounted perpendicular to the LEDs of the optical channel to correct for potential temperature induced drift, e.g. due to warming up of the LED at the beginning of each deployment and environmental temperature changes. The reagent and standard solutions were supplied to the microfluidic channels via PTFE tubing (0.5 mm ID) which connected fluid reservoirs with fluid inlets on the microfluidic chip. The reservoirs (transparent flexible bags, Flexboy-Bag, Sartorius) were kept in a cylindrical PVC tube (200 mm in diameter, 440 mm in length) mounted on top of the analyser’s main housing. The actual design of the analyser (microfluidic diagram) and the data processing procedure to convert the raw PD output into absorbance values are reported in detail in the Supplementary Information.

### Deployment and discrete sampling

**Figure 8:**
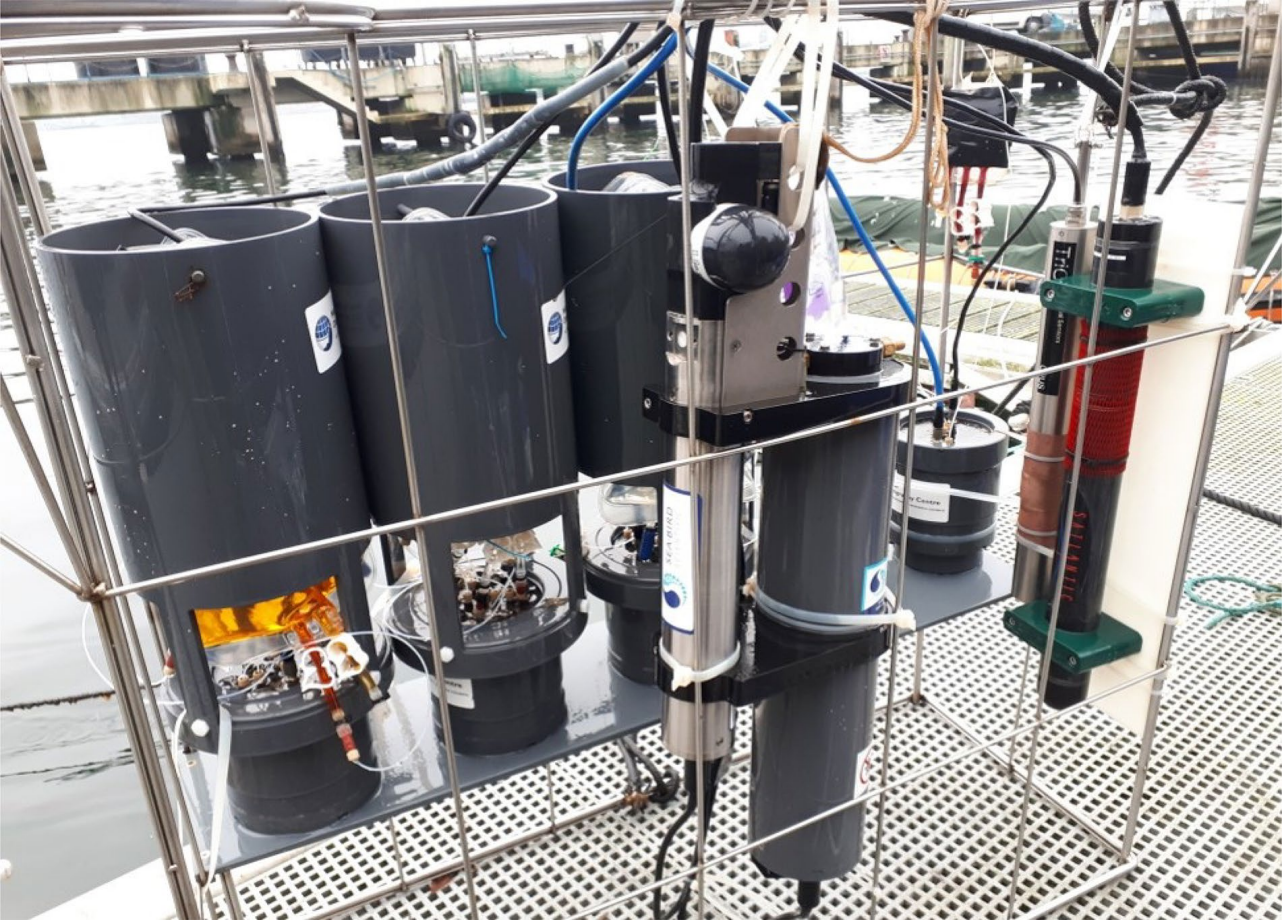
Deployment setup after recovery with the DMn LoC analyser on the left and SeapHOx unit in the front.

The capability of the LoC analyser for DMn measurements under environmental conditions was tested during a field campaign conducted in October/November 2018 in the inner Kiel Fjord (Germany). The analyser was deployed together with other LoC devices (for in situ analysis of Fe(II), DFe, and pH) and a SeapHOx unit (SeaFET pH sensor plus SBE 37-SMP-ODO MicroCAT CTD + DO sensor) for continuous acquisition of hydrographic parameters (pH, temperature, salinity and dissolved oxygen). All instruments were mounted on a frame (Fig. 8) which was lowered from a pontoon to a water depth of 2 m.

The Mn analyser was equipped for the deployment with a blank solution (South Atlantic seawater diluted with de-ionized water), two Mn(II) standard solutions and PAN reagent. Blank and standard solutions were prepared at a salinity of 18 using South Atlantic seawater (with initial DMn concentrations below the analyser's detection limit) in order to closely match the sample matrix of fjord water^41^. The analysis of a sample, which was withdrawn through a 0.22 µm membrane filter (Millipore, polyethersulfone (PES)) from a PTFE tubing with an inlet at 2.0 m depth, was undertaken every 90 min. Prior to each sample measurement, a calibration procedure was conducted using the blank solution followed by analysis of 150 nM and 300 nM Mn(II) standards (for a detailed overview of the measurement routine see Table S1 in the Supplementary Information). In order to validate the DMn concentrations measured by the in situ analyser, discrete samples for trace metal analysis were collected using a peristaltic pump (Masterflex L/S series, Cole-Palmer) up to four times per day, along with samples for dissolved organic carbon (DOC) and macronutrients. A detailed description of discrete sample handling and analysis can be found in the Supplementary Information.

## Supplementary Information

Supplementary Information.

## Data Availability

The data generated in this study are available from the corresponding author upon request.

## References

[1] Twining BS, Baines SB (2013). The trace metal composition of marine phytoplankton. Annu. Rev. Mar. Sci..

[2] Middag R, de Baar HJW, Laan P, Cai PH, van Ooijen JC (2011). Dissolved manganese in the Atlantic sector of the Southern Ocean. Deep Res. Part II Top. Stud. Oceanogr..

[3] Wu M (2019). Manganese and iron deficiency in Southern Ocean *Phaeocystis**antarctica* populations revealed through taxon-specific protein indicators. Nat. Commun..

[4] Pausch F, Bischof K, Trimborn S (2019). Iron and manganese co-limit growth of the Southern Ocean diatom *Chaetoceros**debilis*. PLoS ONE.

[5] Tachikawa K, Handel C, Dupré B (1997). Distribution of rare earth elements and neodymium isotopes in settling particulate material of the tropical Atlantic Ocean (EUMELI site). Deep Sea Res. Part I Oceanogr. Res. Pap..

[6] Sunda WG, Huntsman SA, Harvey GR (1983). Photoreduction of manganese oxides in seawater and its geochemical and biological implications. Nature.

[7] Middag R (2015). Intercomparison of dissolved trace elements at the Bermuda Atlantic Time Series station. Mar. Chem..

[8] Statham PJ, Yeats PA, Landing WM (1998). Manganese in the eastern Atlantic Ocean: Processes influencing deep and surface water distributions. Mar. Chem..

[9] Chin CS (1994). In situ observations of dissolved iron and manganese in hydrothermal vent plumes, Juan de Fuca Ridge. J. Geophys. Res..

[10] Sands CM, Connelly DP, Statham PJ, German CR (2012). Size fractionation of trace metals in the Edmond hydrothermal plume, Central Indian Ocean. Earth Planet. Sci. Lett..

[11] Kremling K, Hydes D (1988). Summer distribution of dissolved Al, Cd Co, Cu, Mn and Ni in surface waters around the British Isles. Cont. Shelf Res..

[12] Statham PJ (2005). Spatially complex distribution of dissolved manganese in a Fjord as revealed by high-resolution in situ sensing using the autonomous underwater vehicle autosub. Environ. Sci. Technol..

[13] Nakashima S, Sturgeon RE, Willie SN, Berman SS (1988). Determination of trace metals in seawater by graphite furnace atomic absorption spectrometry with preconcentration on silica-immobilized 8-hydroxyquinoline in a flow-system. Fresenius’ Z. Anal. Chem..

[14] Rapp I, Schlosser C, Rusiecka D, Gledhill M, Achterberg EP (2017). Automated preconcentration of Fe, Zn, Cu, Ni, Cd, Pb Co, and Mn in seawater with analysis using high-resolution sector field inductively-coupled plasma mass spectrometry. Anal. Chim. Acta.

[15] Otero-Romaní J, Moreda-Piñeiro A, Bermejo-Barrera A, Bermejo-Barrera P (2005). Evaluation of commercial C18 cartridges for trace elements solid phase extraction from seawater followed by inductively coupled plasma-optical emission spectrometry determination. Anal. Chim. Acta.

[16] Beaton AD (2012). Lab-on-chip measurement of nitrate and nitrite for in situ analysis of natural waters. Environ. Sci. Technol..

[17] Grand MM (2017). A lab-on-chip phosphate analyzer for long-term in situ monitoring at fixed observatories: Optimization and performance evaluation in estuarine and oligotrophic coastal waters. Front. Mar. Sci..

[18] Rérolle VMC (2013). Development of a colorimetric microfluidic pH sensor for autonomous seawater measurements. Anal. Chim. Acta.

[19] Coale KH, Chin CS, Massoth GJ, Johnson KS, Baker ET (1991). Insitu chemical mapping of dissolved iron and manganese in hydrothermal plumes. Nature.

[20] Massoth GJ (1998). Manganese and iron in hydrothermal plumes resulting from the 1996 Gorda Ridge event. Deep. Res. Part II Top. Stud. Oceanogr..

[21] Klinkhammer GP (1994). Fiber optic spectrometers for in-situ measurements in the oceans: The ZAPS Probe. Mar. Chem..

[22] Okamura K (2001). Development of a deep-sea in situ Mn analyzer and its application for hydrothermal plume observation. Mar. Chem..

[23] Okamura K (2004). Development of an in situ manganese analyzer using micro-diaphragm pumps and its application to time-series observations in a hydrothermal field at the Suiyo seamount. Geochem. J..

[24] Meyer D (2016). A multi-pumping flow system for in situ measurements of dissolved manganese in aquatic systems. Sensors (Switzerland).

[25] Kremling K (2007). Determination of trace elements. Methods Seawater Anal..

[26] Chiswell B, O’Halloran KR (1991). Comparison of three colorimetric methods for the determination of manganese in freshwaters. Talanta.

[27] Madison AS, Tebo BM, Luther GW (2011). Simultaneous determination of soluble manganese(III), manganese(II) and total manganese in natural (pore)waters. Talanta.

[28] Goto K, Taguchi S, Fukue Y, Ohta K, Watanabe H (1977). Spectrophotometric determination of manganese with 1-(2-pyridylazo)-2-naphthol and a non-ionic surfactant. Talanta.

[29] Geißler F (2017). Evaluation of a ferrozine based autonomous in situ lab-on-chip analyzer for dissolved iron species in coastal waters. Front. Mar. Sci..

[30] Chin CS, Johnson KS, Coale KH (1992). Spectrophotometric determination of dissolved manganese in natural waters with 1-(2-pyridylazo)-2-naphthol: Application to analysis in situ in hydrothermal plumes. Mar. Chem..

[31] Milani A, Statham PJ, Mowlem MC, Connelly DP (2015). Development and application of a microfluidic in-situ analyzer for dissolved Fe and Mn in natural waters. Talanta.

[32] Statham PJ (2003). Mapping the 3D spatial distribution of dissolved manganese in coastal waters using an in situ analyser and the autonomous underwater vehicle Autosub. Underw. Technol..

[33] Skiba M (2016). Development of Microfluidic Pre-concentration System for Metals in Seawater.

[34] Xia F, Cassidy RM (1991). Application of micelles in postcolumn reaction systems. Anal. Chem..

[35] Clinton-Bailey GS (2017). A lab-on-chip analyzer for in situ measurement of soluble reactive phosphate: Improved phosphate blue assay and application to fluvial monitoring. Environ. Sci. Technol..

[36] Evers A, Hancock RD, Martell AE, Motekaitis RJ (1989). Metal ion recognition in ligands with negatively charged oxygen donor groups. Complexation of iron(III), gallium(III), indium(III), aluminum(III), and other highly charged metal ions. Inorg. Chem..

[37] Hernlem BJ, Vane LM, Sayles GD (1996). Stability constants for complexes of the siderophore desferrioxamine B with selected heavy metal cations. Inorg. Chim. Acta.

[38] Duckworth OW, Sposito G (2005). Siderophore-manganese (III) interactions. I. Air-oxidation of manganese(II) promoted by desferrioxamine B. Environ. Sci. Technol..

[39] Gledhill M, Buck KN (2012). The organic complexation of iron in the marine environment: A review. Front. Microbiol..

[40] Rose AL, Waite TD (2003). Kinetics of iron complexation by dissolved natural organic matter in coastal waters. Mar. Chem..

[41] Feng S, Huang Y, Yuan D, Zhu Y, Zhou T (2015). Development and application of a shipboard method for spectrophotometric determination of trace dissolved manganese in estuarine and coastal waters. Cont. Shelf Res..

[42] Sunda WG, Huntsman SA (1988). Effect of sunlight on redox cycles of manganese in the southwestern Sargasso Sea. Deep Sea Res. Part A Oceanogr. Res. Pap..

[43] Roitz JS, Bruland KW (1997). Determination of dissolved manganese(II) in coastal and estuarine waters by differential pulse cathodic stripping voltammetry. Anal. Chim. Acta.

[44] Oldham VE, Mucci A, Tebo BM, Luther GW (2017). Soluble Mn(III)–L complexes are abundant in oxygenated waters and stabilized by humic ligands. Geochim. Cosmochim. Acta.

[45] Burdige DJ (1993). The biogeochemistry of manganese and iron reduction in marine sediments. Earth-Sci. Rev..

[46] Yao W, Millero FJ (1996). Adsorption of phosphate on manganese dioxide in seawater. Environ. Sci. Technol..

[47] Floquet CFA (2011). Nanomolar detection with high sensitivity microfluidic absorption cells manufactured in tinted PMMA for chemical analysis. Talanta.

