# Supplementary material for: Lab-on-chip analyser for the in situ determination of dissolved manganese in seawater
Source: Sci Rep. 2021 Jan 27;11:2382. doi: 10.1038/s41598-021-81779-3 (PMC7840679; doi:10.1038/s41598-021-81779-3)
Supplement: Supplementary file 1 — Supplementary Information. [file 41598_2021_81779_MOESM1_ESM.docx]

# Supplementary Information: Lab-on-chip analyser for the *in situ* determination of dissolved Mn in seawater

Felix Geißler^1^, Eric P. Achterberg^1^, Alexander D. Beaton^2^, Mark J. Hopwood^1^, Mario Esposito^1^, Matt C. Mowlem^2^, Douglas P. Connelly^2^, Douglas Wallace^3^

^1^Chemical Oceanography, Marine Biogeochemistry, GEOMAR Helmholtz Centre for Ocean Research Kiel, Kiel, Germany

^2^National Oceanography Centre, Southampton SO14 3ZH, United Kingdom

^3^Department of Oceanography, Dalhousie University, Halifax, Nova Scotia, Canada

# Chip design, measurement routine and data processing


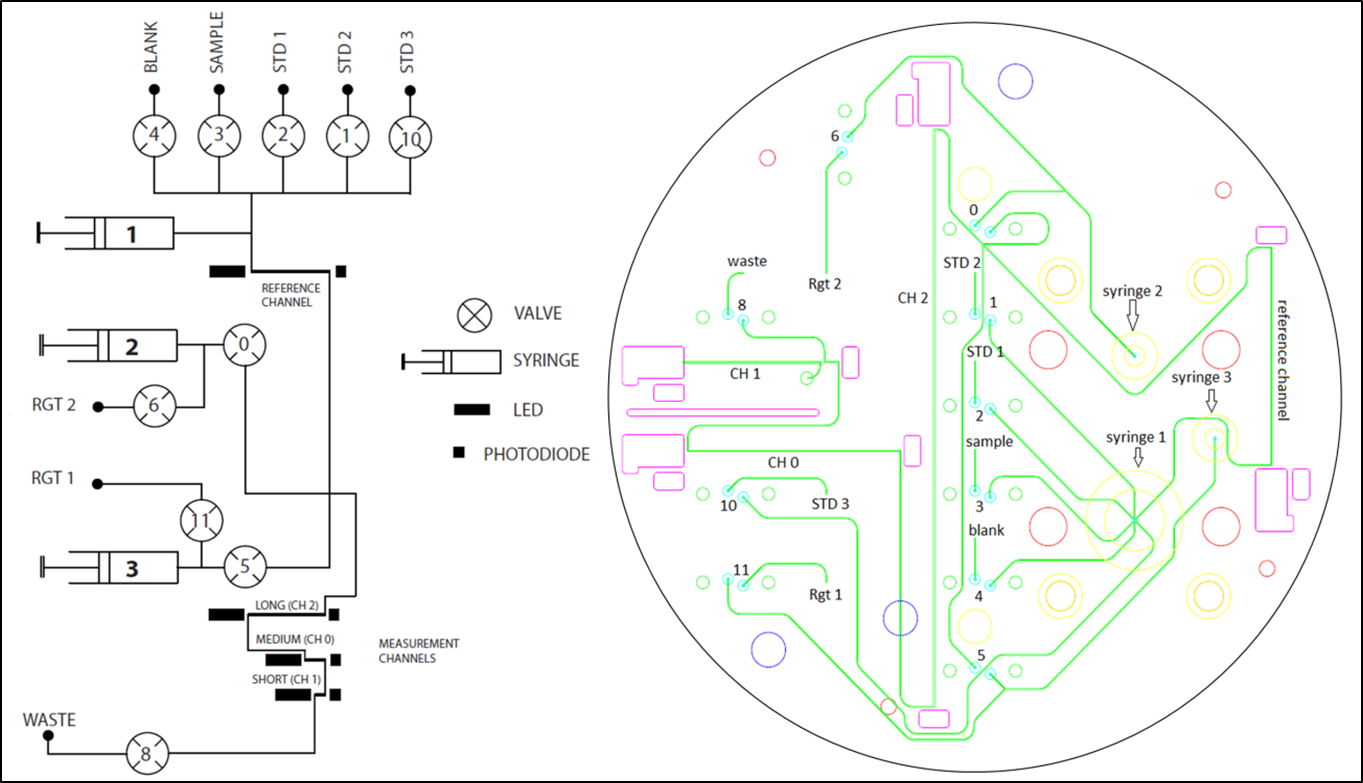
 **Figure S1:** Microfluidic diagram of LoC analyser, simplified (left) and CAD draw of the actual layout (right). Large barrel ((1); ~560 µL) of the syringe pump unit was used for blank, sample and standards, small barrel ((3); ~63 µL) was used for the PAN reagent, ports labelled with ‘STD 3’ and ‘RGT 2’ were spares. Colour code for the CAD draw: green – microfluidic channels, light blue – solenoid valves and fluid thru holes, yellow double circles – syringe pump barrels, pink – LEDs and PDs, all other circles represent filling, mounting, thermistor and communication holes.

**Table S1:** Measurement protocol for a deployment using one blank and two standards for calibration. Numbering of valves according to **Figure S1**.

|  | Step | Step Description | Valves open | Executions |
| --- | --- | --- | --- | --- |
|  | 0 | Waiting for start command |  |  |
| BLANK | 1 | Withdraw Blank and reagent | 4, 6 , 11 | 5x flushed with blank |
|  | 2 | Inject Blank, reagent is pumped back in reservoir | 8, 6, 11 |  |
|  | 3 | Decision state |  |  |
|  | 4 | Withdraw Blank and reagent | 4, 6, 11 |  |
|  | 5 | Reference Blank $\left( V_{BLK}^{R}, I_{BLK}^{R} \right)$ |  |  |
|  | 6 | Inject Blank + reagent | 8, 6, 5 |  |
|  | 7 | Decision state |  |  |
|  | 8 | Waiting period |  |  |
|  | 9 | Measurement state $\left( V_{BLK}, I_{BLK} \right)$ |  |  |
| STANDARD 1 | 10 | Withdraw STD 1 and reagent | 2, 6, 11 | 5x flushed with STD 1 |
|  | 11 | Inject STD 1, reagent is pumped back in reservoir | 8, 6, 11 |  |
|  | 12 | Decision state |  |  |
|  | 13 | Withdraw STD 1 and reagent | 2, 6, 11 |  |
|  | 14 | Reference STD1 $\left( V_{STD1}^{R} , I_{STD1}^{R} \right)$ |  |  |
|  | 15 | Inject STD 1 and reagent | 8, 6, 5 |  |
|  | 16 | Decision state |  |  |
|  | 17 | Waiting period |  |  |
|  | 18 | Measurement state $\left( V_{STD1}, I_{STD1} \right)$ |  |  |
| STANDARD 2 | 19 | Withdraw STD 2 and reagent | 1, 6, 11 | 5x flushed with STD 2 |
|  | 20 | Inject STD 2, reagent is pumped back in reservoir | 8, 6, 11 |  |
|  | 21 | Decision state |  |  |
|  | 22 | Withdraw STD 2 and reagent | 1, 6, 11 |  |
|  | 23 | Reference STD 2 $\left( V_{STD2}^{R}, I_{STD2}^{R} \right)$ |  |  |
|  | 24 | Inject STD 2 and reagent | 8, 6, 5 |  |
|  | 25 | Decision state |  |  |
|  | 26 | Waiting period |  |  |
|  | 27 | Measurement state $\left( V_{STD2}, I_{STD2} \right)$ |  |  |
|  | 28 | Withdraw Sample and reagent | 3, 6, 11 | 5x flushed with sample |
|  | 29 | Inject Sample, reagent is pumped back in reservoir | 8, 6, 11 |  |
| SAMPLE | 30 | Decision state |  |  |
|  | 31 | Withdraw Sample and reagent | 3, 6, 11 |  |
|  | 32 | Reference Sample $\left( V_{Sample}^{R}, I_{Sample}^{R} \right)$ |  |  |
|  | 33 | Inject Sample and reagent | 8, 6, 5 |  |
|  | 34 | Decision state |  |  |
|  | 35 | Waiting period |  |  |
|  | 36 | Measurement state $\left( V_{Sample}, I_{Sample} \right)$ |  |  |

The raw output of the PDs for each measurement was converted into absorbance values according to equation (1).

| $Absorbance=-\log_{10} \left( \frac{V_{Sample}}{V_{Sample}^{R}}\cdot\frac{I_{Sample}^{R}}{I_{Sample}} \right)$ | (1) |
| --- | --- |

A linear fit between the absorbance values of the blank and the standard solutions was then applied as calibration curve on the absorbance of the sample to calculate the present DMn concentration. For each analysis the system was flushed five times with the respective blank, standard or sample without PAN reagent to minimize carry over effects of the previous solution in the system. Such a flushing procedure was verified for the measurement of Fe ^1^ in order to remove any remaining sample from the cell. At the end of each flushing procedure, the mean five second output of the PD was recorded to compensate for any matrix effects ($V_{Sample}^{R}$ in equation (1)). After the flushing procedure the blank, standard or sample was injected together with the PAN reagent. A waiting time of 15 min was applied under stopped flow condition in order to allow mixing, chemical reaction and full colour development. The five second mean of the raw PD output was then used, as $V_{Sample}$ in equation (1), for the calculation of the respective absorbance value. As the illumination intensity of the emitted light from the LEDs varies with temperature a scaling factor ${I_{Sample}^{R}}/{I_{Sample}}$ is integrated in equation (1). Here, $I_{Sample}^{R}$ and $I_{Sample}$ are the raw data of the monitoring PDs after the flushing procedure and after full color development, respectively.

# Discrete samples

In order to validate the DMn concentrations measured during the field deployment of the *in situ* analyser, discrete samples for trace metal analysis were collected up to four times per day using a peristaltic pump (Masterflex L/S series, Cole-Palmer) with an acid cleaned 6.4 mm ID C-Flex tubing (Masterflex, Cole-Palmer) attached. Manual sample collection was timed according to the sample routine of the analyser such that each manual event corresponded to an analyser-derived measurement. The inlet of the tubing was attached to the stainless steel frame at the same height as the analyser’s input. A 0.2 µm poly(ether sulfone) (PES) filter capsule including a 0.8 µm pre-filter (AcroPak 500, Pall GmbH) was used for filtration of the discrete samples directly at the sampling site. Samples for dissolved trace metal analysis were collected in cleaned 125 mL LDPE Nalgene bottles and acidified in a clean laboratory with 180 µL concentrated HCl (ultra-pure acid grade, ROMIL) to pH < 2. After three months of storage dissolved trace metal samples were diluted 1:10 using 1 M distilled HNO_3_ (Spa grade, ROMIL, distilled using a sub-boiling PFA distillation system) and analysed by high resolution ICP-MS (ELEMENT XR, ThermoFisherScientific). Analysis of NASS-7 and CASS-6 certified reference materials via the same method yielded Mn concentrations of 14.36 ± 0.41 nM (certified 13.65 ± 1.09 nM) and 36.5 ± 1.2 nM (certified 40.4 ± 2.2 nM), respectively. Pre-combusted 20 mL glass vials were used for the collection of dissolved organic carbon (DOC) samples. Sample collection was followed by acidification with 25 µL of concentrated HCl (ultra purity acid grade, ROMIL). Analysis was performed as non-purgeable organic carbon (NPOC) using a high temperature catalytic combustion approach (Shimadzu TOC-L CPH). Samples for macronutrients were collected in 15 mL Falcon centrifuge tubes and kept frozen until analysis using a segmented flow autoanalyzer (QuAAtro, SEAL Analytical).

# References

1. Geißler, F. *et al.* Evaluation of a Ferrozine Based Autonomous in Situ Lab-on-Chip Analyzer for Dissolved Iron Species in Coastal Waters. *Front. Mar. Sci.* **4**, (2017).
